# Disentangling decision errors from action execution in mouse-tracking studies: The case of effect-based action control

**DOI:** 10.3758/s13414-024-02974-8

**Published:** 2024-11-20

**Authors:** Solveig Tonn, Moritz Schaaf, Wilfried Kunde, Roland Pfister

**Affiliations:** 1 Department of Psychology, University of Trier, Johanniterufer 15, 54290 Trier, Germany; 2 Department of Psychology, University of Würzburg, Würzburg, Germany

**Keywords:** Mouse-tracking, Response-effect compatibility, Ideomotor framework, Action execution, Initial decision errors

## Abstract

Mouse-tracking is regarded as a powerful technique to investigate latent cognitive and emotional states. However, drawing inferences from this manifold data source carries the risk of several pitfalls, especially when using aggregated data rather than single-trial trajectories. Researchers might reach wrong conclusions because averages lump together two distinct contributions that speak towards fundamentally different mechanisms underlying between-condition differences: influences from online-processing during action execution and influences from incomplete decision processes. Here, we propose a simple method to assess these factors, thus allowing us to probe whether process-pure interpretations are appropriate. By applying this method to data from 12 published experiments on ideomotor action control, we show that the interpretation of previous results changes when dissociating online processing from decision and initiation errors. Researchers using mouse-tracking to investigate cognition and emotion are therefore well advised to conduct detailed trial-by-trial analyses, particularly when they test for direct leakage of ongoing processing into movement trajectories.

## Introduction

Motor actions comprise more than a sequence of movements. They are driven by certain intentions and involve manifold cognitive and emotional processes. As recent observations suggest that the intention behind an action heavily influences the kinematics of the ensuing movement, the analysis of unfolding motions is regarded as a powerful tool to shed light on cognitive and emotional processes (Ansuini et al., 2014; Georgiou et al., 2007; Sartori et al., 2011; Song & Nakayama, 2009; Stillman et al., 2018). This potential has attracted the attention of behavioral scientists, especially in cognitive psychology, a field with a long history of employing reaction-time setups to test for the speed of processing. While conventional, chronometric setups yield essentially one data point per correct response, movement trajectories are able to provide multiple data points for every movement, substantially increasing the amount of information that can be analyzed (Bundt et al., 2018; Fischer & Hartmann, 2014; Maldonado et al., 2019; Zgonnikov et al., 2017).

One particular prominent way to capture movement trajectories is the simple and elegant means of logging the coordinates of a mouse cursor across time (Freeman & Ambady, 2009; McKinstry et al., 2008). Mouse-tracking has generated valuable insights in numerous fields, including social categorization (Dale et al., 2007; Hehman et al., 2014; Lazerus et al., 2016; Stolier & Freeman, 2017), self-control in decision-making (Buttlar & Walther, 2019; O’Hora et al., 2016; Stillman et al., 2017, 2018), and semantic processing (Dale & Duran, 2011; Spivey et al., 2005; Wirth et al., 2019). Further, it has been employed to investigate motivational topics like approach and avoidance tendencies (Boschet et al., 2022; Dignath et al., 2014, 2020; Wirth et al., 2016), rule-breaking (Jusyte et al., 2017; Pfister et al., 2016; Wirth et al., 2018), and cognitive conflict (Boschet et al., 2022; Erb et al., 2016; Mittelstädt et al., 2023; Quétard et al., 2023; Scherbaum et al., 2010).

Despite its widespread use, comprehensive standards for experimental design and especially data analysis have not yet been established. Mouse-tracking experiments differ, for example, regarding their starting procedure (dynamic vs. static), response mode (terminating a response by clicking vs. reaching a target), or cursor speed (Kieslich et al., 2018; Scherbaum & Kieslich, 2018). In fact, recent observations suggest that these seemingly slight differences in the experimental procedure can lead to puzzling differences in observed results and corresponding conclusions (Grage et al., 2019; Kieslich et al., 2020; Schoemann et al., 2019, 2021; Wirth et al., 2020).

The only aspect of mouse-tracking analyses that enjoys widespread consensus is the use of aggregated means instead of individual trajectories to plot experimental results (Buttlar & Walther, 2019; Dieciuc et al., 2019; Pfister et al., 2016; Stillman et al., 2018; Ye & Damian, 2022). Ironically, this consensus comes with major limitations as it neglects that one and the same average trajectory can derive from highly different trajectories on a by-participant or by-trial level (e.g., Matejka & Fitzmaurice, 2017).

### Single movements versus aggregate statistics

The observation that identical trajectory averages can result from profoundly different individual movements calls for methods to evaluate different trajectory types. Such methods include the classification via cluster-analytical methods (Wulff et al., 2018) or graphical approaches such as heatmaps of individual movements (Garcia-Guerrero et al., 2022; Scherbaum et al., 2010; Vogel et al., 2018). These highly sophisticated methods have not been adopted widely, however.

Thus, we suggest an easy-to-implement method that aims to reach a similar goal. Our approach provides a straightforward way to assess the particularly relevant distinction of smooth, single-step movements versus multi-step movements in which an initial movement is re-evaluated and then revised along the movement trajectory. Single-step and multi-step movements clearly involve different cognitive processes and, therefore, this distinction must be considered when interpreting the results of an experiment (see van der Wel et al., 2009 and Spivey et al., 2010, for a conceptually related discussion of discrete vs. continuous influences on movement trajectories in a lexical decision task).

### A simple criterion

Multi-step movements often come in the shape of initial movements in the wrong direction that are corrected by pausing and changing direction along the way. Such movements reflect errors in the initial decision of where to move, and should therefore not be considered when movement execution (rather than decision making) is targeted by an experiment. Although statistical clustering (Wulff et al., 2018) is the currently most elegant and sophisticated method to distinguish different types of movements, it may not always be ideal for two reasons. First, it does not take the experimental design into account. This includes the display geometry in terms of common home and target areas that define a movement’s start and endpoint. Second, current clustering algorithms are inherently spatial, whereas multi-step movements may also come as stop-and-go movements with breaks or decelerations along the trajectory (Dale & Duran, 2011; Fishbach et al., 2005, 2007; Kieffaber et al., 2023).

We therefore suggest combining a simple spatial criterion relating to initial decision errors with a validation of this criterion in terms of a movement’s corresponding velocity. As a spatial cutoff, we suggest using a simple vertical cutoff line based on the display geometry of a given study. This method allows to easily exclude movements with wrong initial decisions. Doing so focuses the analyses on trials with completed decisions before movement start. As a spatio-temporal method, we suggest using velocity plots to assess whether trajectories on different sides of the spatial cutoff do indeed come with different attributes.^1^

### A topical example: Effect-based action control

To demonstrate the combined application of both strategies and to document the importance of distinguishing between smooth single-step movements versus multi-step movements, we re-evaluated a set of findings from a particular experimental design – the response-effect compatibility paradigm (Kunde, 2001; Pfister et al., 2014). We chose this specific paradigm for two reasons: First, we prefer to criticize (and potentially deconstruct) our own previous work rather than the work of others. Second, and more importantly, this area of research has its historical origin in theories of motor control (Harleß, 1862; Herbart, 1825; James, 1890; for historical comments, see Pfister & Janczyk, 2012; Stock & Stock, 2004), not in theories of decision making. As a result, investigators utilized mouse-tracking to derive conclusions about action execution rather than decision making. This aim is, of course, not applicable to all research using mouse-tracking. As we outline in the *Discussion*, other research domains are particularly interested in movements with non-completed decisions upon movement start (e.g., Boschet et al., 2022; Dale & Duran, 2011; Dshemuchadse et al., 2013). Instead of disregarding these movements, such studies might intentionally utilize experimental designs evoking movements with incomplete decisions. If, however, the underlying question is whether effect anticipations influence responses even beyond the categorical selection of an action goal (Pfister et al., 2014), excluding influences from (partial) errors is pivotal.

**Fig. 1:**
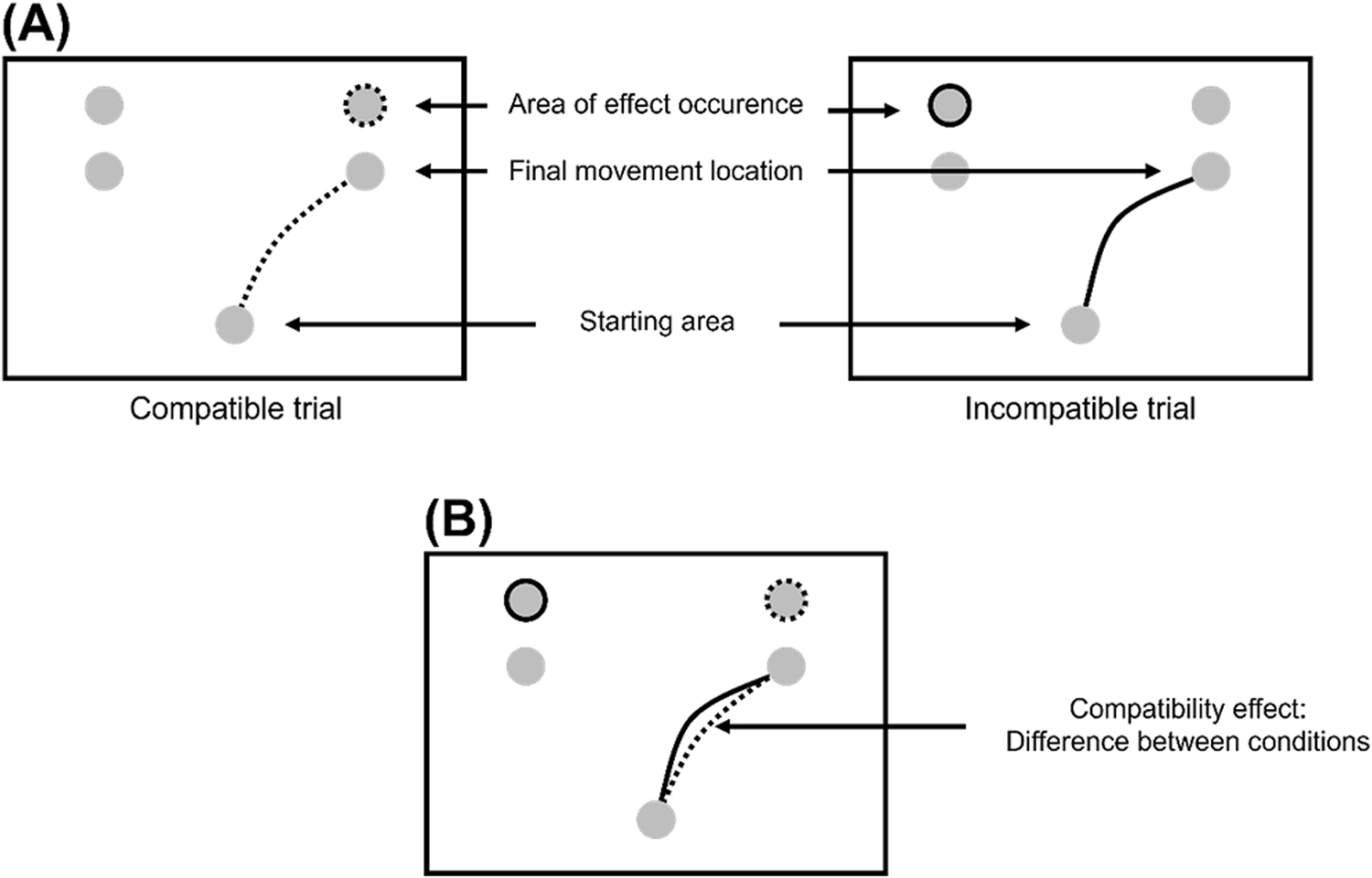
Experimental design and previously observed data pattern. (**A**) Movements began in a starting area in the lower middle of the screen and ended in one of two possible final movement locations. Depending on the response-effect compatibility mapping, action effects either occurred on the same side as the final movement location (dotted circle) or on the respective other side (solid circle). (**B**) The previously observed data pattern: Movements were biased towards the anticipated effect location, resulting in a more curved movement trajectory with incompatible effects (solid trajectory) compared to compatible effects (dotted trajectory)

Studies using the response-effect compatibility design typically assess the content of action representations by coupling actions with effects that share or oppose characteristics of the action. In other words, these experiments introduce feature overlap (dimensional overlap; Kornblum et al., 1990) between responses (i.e., body movements) and response-contingent perceptual effects (e.g., in the agent’s environment). In the case of mouse-tracking, this common feature is usually manipulated through a spatial left-versus-right arrangement of movement targets and the visual effects that are triggered by reaching a target (Fig. 1; see Pfister et al., 2014; Schonard et al., 2021). These effects can be compatible (i.e., when a movement to the right evokes a visual effect on the right-hand side) or they can be incompatible (i.e., when a movement to the right evokes a visual effect on the hand side). Crucially, previous studies observed ongoing movements to be attracted towards the movement-contingent effect. Put differently, if the completion of movements triggered an effect on the other side of the screen, movements deviated more to that side than when they triggered an effect on the same side. This result was interpreted as evidence suggesting that the visual effect was anticipated during motor execution and, thus, plays a pivotal role for the control of efferent activity. That is, these studies specifically sought to investigate influences on action execution, and regarded spatial aspects of the trajectory as “postselection measures” (Hommel et al., 2017, p. 825).

In-depth analyses of a recent experiment, however, challenged this interpretation by showing that such compatibility effects may derive from a subset of movements with initial decision errors (Tonn et al., 2023). In fact, excluding these movements made the response-effect compatibility effect disappear in spatial measures. Therefore, re-evaluating previous evidence (and previous interpretations) from this particular paradigm is ideal as a first estimate of how prevalent and pervasive the influence of multi-step movements is.

## Method

All mouse-tracking experiments that were included in the present re-analyses investigated the influence of action effects on action execution (Hommel et al., 2017; Pfister et al., 2014; Schonard et al., 2021; Tonn et al., 2023; Wirth et al., 2015).

### Experimental design

The common denominator of all analyzed studies is that they used a setup with five relevant areas, as shown in Fig. 1: A home area at the bottom center, two target areas in the upper left and right corner, and two effect locations directly above the target areas. Before the start of each trial, participants were informed about the current mapping of their own responses to the ensuing effects (compatible vs. incompatible) through visual cues in the target areas. In other words, compatibility was varied trial-wise, and participants could prepare for the upcoming response-effect relation. After signaling that they were ready for the current trial by moving to the home area, an imperative stimulus instructed participants to produce an effect at either the left or the right location. This required a movement to the target area directly below the desired effect location in the compatible condition whereas it required a movement to the respective other target area in the incompatible condition. While participants were instructed to execute the task as quickly and accurately as possible, no explicit movement initiation deadline was implemented. The underlying rationale for this design decision was to ensure that participants started the movement only after completing their decision. Consequently, to isolate influences on movement execution, the analyzed trajectories were truncated to the part between leaving the starting area and reaching the target area. All experiments assessed how anticipating an action effect shapes action execution (see Fig. 1B), that is, whether movements are systematically biased towards the location of their ensuing effect. With effect sizes ranging from *d*_*z*_ = 0.38 to *d*_*z*_ = 1.38, all experiments consistently showed that incompatible movements were more curved than compatible movements. However, we argue here that these effects were mainly driven by incomplete decisions in a fraction of the trials.

### Analyses

#### Basic approach

For analyzing mouse-movement trajectories, we used the R package *mousetRajectory* (Pfister et al., 2024). We extracted initiation time (IT), movement time (MT), area under the curve (AUC), and maximum absolute deviation (MAD) from the individual trajectories. IT was measured as the time from the onset of the imperative stimulus until the cursor left the starting area. MT was measured from this point in time until the cursor arrived at the target area. AUCs were computed as the (signed) area between the executed and the optimal path (straight line through start and end coordinates), and MADs were computed as the (signed) maximum orthogonal deviation of the executed path from the optimal path. Deviations towards the opposite target area were counted as positive, whereas deviations in the other direction were counted as negative. All movements were flipped to the right, the coordinates of each trial were time-normalized and re-sampled with linear interpolation before computing AUCs and MADs, and the resulting normalized trajectories were used for plotting.

For our re-analyses, we used a consistent approach across studies in terms of preprocessing and outlier correction, which naturally leads to minor differences between the re-analyses and the original results: For all analyses, we omitted trials with downward movements of the mouse, trials with errors, and outlier trials. Outliers were defined as trials where any of the four measures deviated more than 2.5 *SD*s from the corresponding cell mean, computed separately for each participant and condition. For brevity, we report only the effect of compatibility, and omit the influences of all other experimental factors (i.e., we report *F*-statistics for the main effect of compatibility in multifactorial designs and *t*-statistics in unifactorial designs). Full data sets and analysis scripts are available via the Open Science Framework at: https://osf.io/hrpk6.

#### Trial-level analyses

All studies reported a consistent impact of upcoming action effects on movement trajectories which had previously been taken to suggest an important role of action effect representations for motor control (Hommel et al., 2017; Pfister et al., 2014; Schonard et al., 2021; Wirth et al., 2015). The suggested spatial criterion for detecting multi-step movements (including initial decision errors) provides a simple and elegant tool to assess the truth value of this interpretation.

**Fig. 2:**
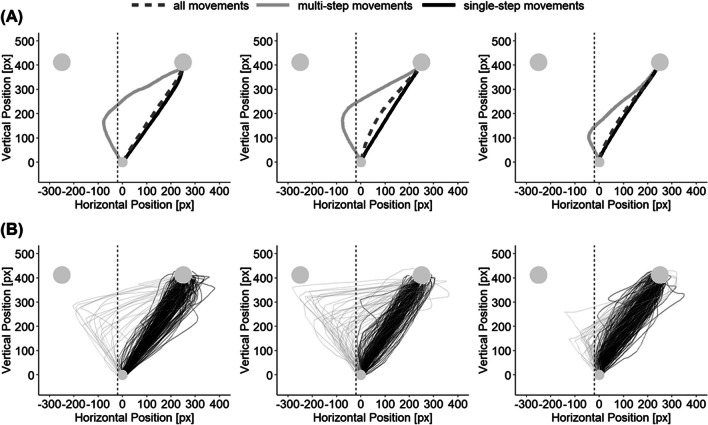
Visualization of the cutoff-criterion. Data from three example participants. (**A**) Average trajectories over all trajectories (dashed, dark gray lines), over trajectories excluded by the cutoff criterion (solid, light gray lines), and over trajectories surviving the cutoff criterion (solid, black lines). (**B**) Individual movements going into the averages trajectories displayed in panel A. The vertical line visualizes the cutoff criterion

Therefore, we separated all movements previously classified as correct (i.e., movements ending on the correct target area) into two groups: Movements directly starting towards the correct target and movements first starting to the wrong side and later changing the course of movement. This excludes movements with initially wrong decisions and puts increased focus on data points with completed decisions upon movement start. The question was whether previously observed effects persist when only taking these latter movements into account. Our classification was implemented by excluding all movements with x-values going below the lowest x-value of the starting area, resulting in a vertical cutoff line touching the starting area on the left (see Fig. 2). This criterion classified more incompatible than compatible trials as multi-step movements, which is consistent with response-time studies reporting more commission errors with incompatible action-effect mappings than with compatible mappings (e.g., Kunde, 2001).

**Fig. 3:**
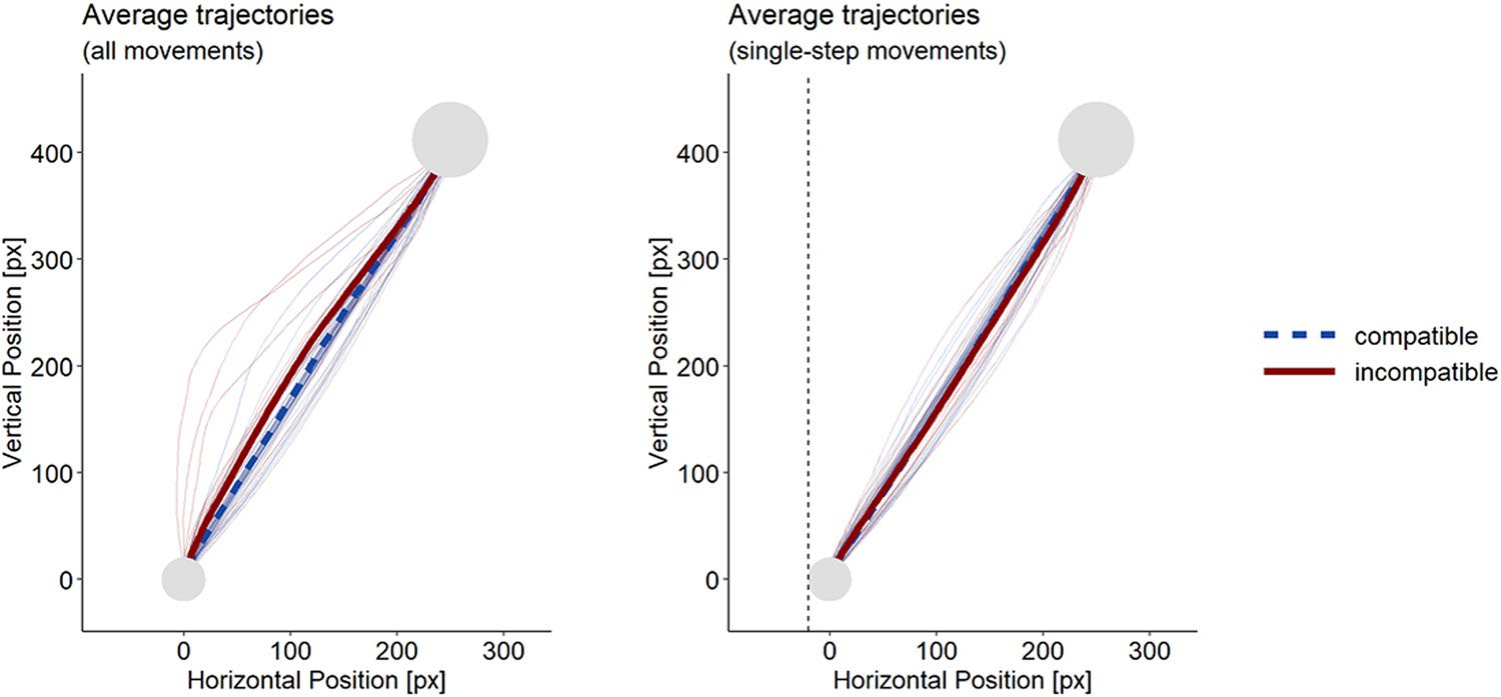
Average trajectories for one exemplary experiment (Pfister et al., 2014, Exp. 2). Average trajectories when using all movements (left) and when excluding multi-step movements (right). Although participant averages (thin lines) are smooth and do not cross our exclusion criterion, the response-effect compatibility effect in spatial measures completely vanishes when multi-step movement trials are excluded: Compatible (blue) and incompatible (red) average trajectories overlap for single-step movements

Using one representative experiment, Fig. 3 demonstrates the impact of this cutoff by showing how average trajectories – as commonly used to visualize experimental results – change when multi-step movements are excluded.

Second, we show for each experiment how inferential statistics change, that is, how the effect evolves, when the criterion is used and then relaxed from its original location (i.e., on the left side of the starting area) up to the middle of the wrong target area. In other words, we plot the standardized effect size of the compatibility effect in the AUC as a function of this cutoff criterion.

**Fig. 4:**
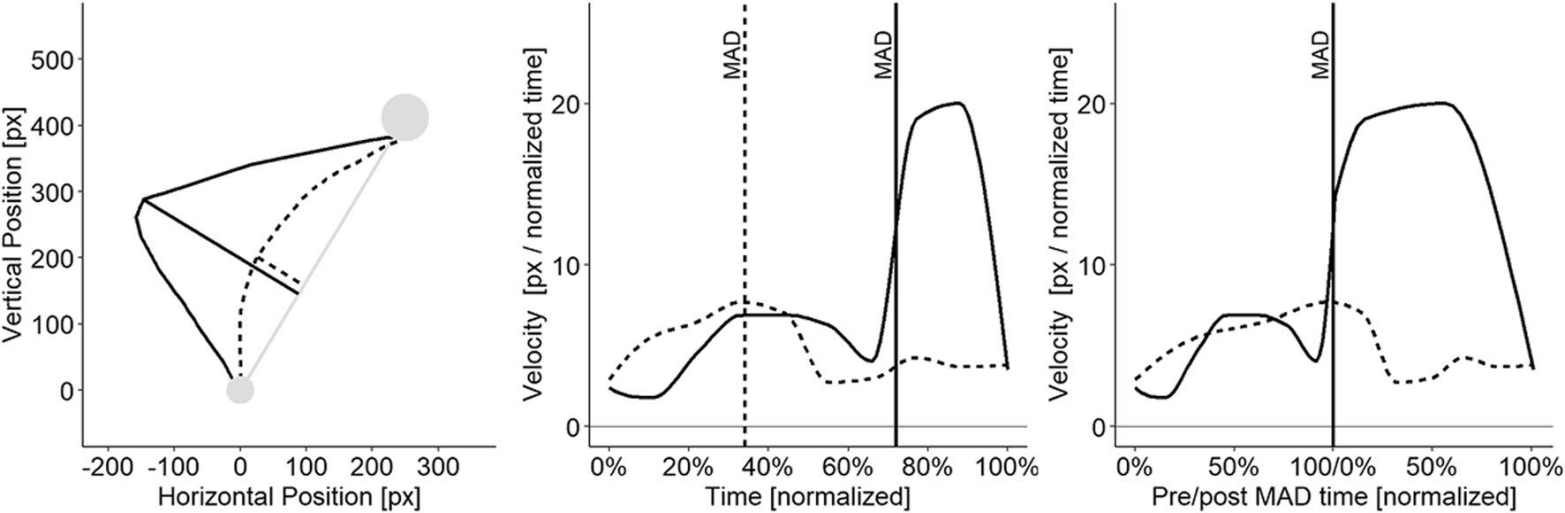
Explanation of the velocity profile plots. For all (time-normalized) trajectories, the timepoint of maximal orthogonal deviation (MAD) from the ideal line is computed (**left**). Trajectories are then separated into pre- and post-MAD parts (**middle**). The resulting sub-trajectories are time-normalized again before velocities can be averaged (**right**). The solid and dashed lines illustrate two exemplary, individual trajectories. Note that even if two movements exhibit their MAD at a similar point on the ideal line (**left**), the timepoint of the MAD might vary substantially (**middle**), necessitating a per-movement, temporal alignment of the velocities (**right**)

As a further check for our spatial criterion, we provide corresponding velocity information for the two different movement types for every experiment via velocity profile plots. To account for variability in the timing of decision changes, we identified the point of maximum absolute deviation within each trajectory and time-normalized velocities from the start of the movement up to this timepoint as well as from this timepoint to the end of the movement (see Fig. 4 for an explanation of this novel procedure).

## Results

Table 1 provides a summary over all re-analyzed experiments, highlighting how the results change when multi-step movements are excluded with the proposed cutoff criterion. Due to consistency in the pattern of results across all experiments, we textually describe only one re-analysis in detail in the main text. Detailed descriptions and visualizations of the other experiments are available in the Appendix. We focus on the Experiment 2 of Pfister et al. (2014) because it served as the design template for all subsequent studies investigating response-effect compatibility effects using mouse-tracking.

**Table 1:** Overview of the re-analyzed experiments

Exp.	Goal of the experiment	Experimental manipulations	Number of trials^a^	DV	REC effect without application of the cutoff criterion^b^	REC effect with application of the cutoff criterion^b^
Pfister et al. (2014)						
Exp. 1	First experiment that investigated REC with mouse-tracking	REC	224	AUC	*t*(19) = 2.75, *p* = .013, *d*_*z*_ = 0.61 (15.8 × 10^3^ vs. 16.9 × 10^3^ px^2^)	*t*(19) = 1.89, *p* = .073, *d*_*z*_ = 0.42
				MAD	*t*(19) = 2.58, *p* = .018, *d*_*z*_ = 0.58 (64.1 vs. 68.1 px)	*t*(19) = 1.17, *p* = .258, *d*_*z*_ = 0.26
				IT	*t*(19) = 2.33, *p* = .031, *d*_*z*_ = 0.52 (652 vs. 672 ms)	*t*(19) = 2.17, *p* = .043, *d*_*z*_ = 0.49 (654 vs. 672 ms)
				MT	*t*(19) = 3.17, *p* = .005, *d*_*z*_ = 0.71 (590 vs. 628 ms)	*t*(19) = 2.77, *p* = .012, *d*_*z*_ = 0.62 (578 vs. 598 ms)
				%CC	*t*(19) = 3.19, *p* = .005, *d*_*z*_ = 0.71 (5.9% vs. 10.3%)	
Exp. 2	In contrast to Exp. 1, participants did not have to start their movement upwards and thus, were able to take the direct path	REC	224	AUC	*t*(19) = 3.59, *p* = .002, *d*_*z*_ = 0.80 (-0.1 × 10^3^ vs. 2.7 × 10^3^ px^2^)	|*t*| < 1
				MAD	*t*(19) = 3.94, *p* = .001, *d*_*z*_ = 0.88 (0.8 vs. 15.6 px)	|*t*| < 1
				IT	*t*(19) = 5.81, *p* < .001, *d*_*z*_ = 1.30 (639 vs. 693 ms)	*t*(19) = 6.31, *p* < .001, *d*_*z*_ = 1.41 (634 vs. 706 ms)
				MT	*t*(19) = 3.12, *p* = .006, *d*_*z*_ = 0.70 (421 vs. 457 ms)	|*t*| < 1
				%CC	*t*(19) = 4.04, *p* = .001, *d*_*z*_ = 0.90 (9.8% vs. 17.5%)	
Wirth et al. (2015)						
Exp. 1	Where is the locus of the mouse-tracking REC effect? Dual tasking, “locus of slack” logic Task 1: pitch discrimination Task 2: REC with mouse-tracking	REC × stimulus onset asynchrony	348	AUC	*F*(1, 15) = 7.63, *p* = .015, η_p_^2^ = .34 (0.7 × 10^3^ vs. 2.8 × 10^3^ px^2^)	*F* < 1
				MAD	*F*(1, 15) = 8.19, *p* = .012, η_p_^2^ = .35 (4.2 vs. 15.0 px)	*F* < 1
				IT	*F*(1, 15) = 26.50, *p* < .001, η_p_^2^ = .64 (880 vs. 950 ms)	*F*(1, 15) = 27.17, *p* < .001, η_p_^2^ = .64 (879 vs. 950 ms)
				MT	*F*(1, 15) = 13.58, *p* = .002, η_p_^2^ = .48 (375 vs. 399 ms)	*F*(1, 15) = 1.70, *p* = .212, η_p_^2^ = .10
				%CC	*t*(15) = 4.30, *p* = .001, *d*_*z*_ = 1.08 (9.4% vs. 17.1%)	
Exp. 2	Dual tasking, “effect propagation” logic: Task 1: REC with mouse-tracking Task 2: pitch discrimination	REC × stimulus onset asynchrony	348	AUC	*F*(1, 15) = 4.53, *p* = .050, η_p_^2^ = .23	*F* < 1
				MAD	*F*(1, 15) = 6.40, *p* = .023, η_p_^2^ = .30 (22.8 vs. 27.9 px)	*F*(1, 15) = 1.00, *p* = .334, η_p_^2^ = .06
				IT	*F*(1, 15) = 3.55, *p* = .079, η_p_^2^ = .19	*F*(1, 15) = 3.87, *p* = .068, η_p_^2^ = .21
				MT	*F*(1, 15) = 9.78,* p* = .007, η_p_^2^ = .39 (1015 vs. 1091 ms)	*F*(1, 15) = 7.66, *p* = .014, η_p_^2^ = .34 (988 vs. 1053 ms)
				%CC	*t*(15) = 2.26, *p* = .039, *d*_*z*_ = 0.56 (13.0% vs. 16.6%)	
Hommel et al. (2017)						
Exp. 1	Do REC effects stem from sensory or affective compatibility? Is this differentially affected by whether actions are free vs. forced choice?	sensory REC^c^ × affective REC^c^ × free vs. forced choice	240	AUC	*F*(1, 34) = 10.41, *p* = .003, η_p_^2^ = .23 (3.2 × 10^3^ vs. 5.3 × 10^3^ px^2^)	*F*(1, 34) = 5.45, *p* = .026, η_p_^2^ = .14 (-0.7 × 10^3^ vs. 0.3 × 10^3^ px^2^)
				MAD	*F*(1, 34) = 9.77, *p* = .004, η_p_^2^ = .22 (17.7 vs. 27.6 px)	*F*(1, 34) = 4.76,* p* = .036*,* η_p_^2^ = .12 (-2.4 vs. 1.5 px)
				IT	*F*(1, 34) = 7.19, *p* = .011, η_p_^2^ = .17 (496 vs. 507 ms)	*F*(1, 34) = 5.38, *p* = .027, η_p_^2^ = .14 (497 vs. 507 ms)
				MT	*F*(1, 34) = 5.69, *p* = .023, η_p_^2^ = .14 (339 vs. 347 ms)	*F*(1, 34) = 5.24, *p* = .028, η_p_^2^ = .13 (317 vs. 324 ms)
				%CC	*t*(34) = 4.82, *p* < .001, *d*_*z*_ = 0.81 (16.5% vs. 22.3%)	
Schonard et al. (2021)						
Exp. 1	Can mouse-tracking REC effects be replicated in a simplified setting?	REC × free vs. forced choice	240	AUC	*F*(1, 19) = 23.22,* p* < .001, η_p_^2^ = .55 (22.7 × 10^3^ vs. 33.7 × 10^3^ px^2^)	*F*(1, 19) = 3.05,* p* = .097*,* η_p_^2^ = .14
				MAD	*F*(1, 19) = 23.00,* p* < .001, η_p_^2^ = .55 (32.4 vs. 51.7 px)	*F*(1, 19) = 3.02,* p* = .098*,* η_p_^2^ = .14
				IT	*F*(1, 19) = 3.77, *p* = .067, η_p_^2^ = .17	*F*(1, 19) = 3.50, *p* = .077, η_p_^2^ = .16
				MT	*F*(1, 19) = 30.65,* p* < .001, η_p_^2^ = .62 (544 vs. 572 ms)	*F*(1, 19) = 6.43, *p* = .020, η_p_^2^ = .25 (535 vs. 552 ms)
				%CC	*t*(19) = 5.52, *p* < .001, *d*_*z*_ = 1.23 (7.1% vs. 16.1%)	
Exp. 2	Is the mouse-tracking REC effect subject to sequential modulation?	REC × previous REC^d^	240	AUC	*t*(39) = 4.37, *p* < .001, *d*_*z*_ = 0.69 (63.2 × 10^3^ vs. 72.7 × 10^3^ px^2^)	*t*(39) = 1.02, *p* = .312, *d*_*z*_ = 0.16
				MAD	*t*(39) = 4.22, *p* < .001,* d*_*z*_ = 0.67 (92.5 vs. 108.1 px)	|*t*| < 1
				IT	*t*(39) = 5.15, *p* < .001, *d*_*z*_ = 0.81 (602 vs. 645 ms)	*t*(39) = 5.80, *p* < .001, *d*_*z*_ = 0.92 (604 vs. 658 ms)
				MT	*t*(39) = 4.75, *p* < .001, *d*_*z*_ = 0.75 (633 vs. 661 ms)	*t*(39) = 3.14, *p* = .003, *d*_*z*_ = 0.50 (613 vs. 628 ms)
				%CC	*t*(39) = 5.11, *p* < .001, *d*_*z*_ = 0.81 (11.1% vs. 17.4%)	
Exp. 3	Excludes dimensional overlap between stimuli and effects	REC × free vs. forced choice	240	AUC	*F*(1, 39) = 8.89, *p* = .005, η_p_^2^ = .19 (13.8 × 10^3^ vs. 19.3 × 10^3^ px^2^)	*F*(1, 39) = 5.42, *p* = .025, η_p_^2^ =.12 (7.2 × 10^3^ vs. 9.7 × 10^3^ px^2^)
				MAD	*F*(1, 39) = 8.63,* p* = .006, η_p_^2^ = .18 (20.7 vs. 30.3 px)	*F*(1, 39) = 4.94,* p* = .032, η_p_^2^ = .11 (9.5 vs. 13.0 px)
				IT	*F*(1, 39) = 10.66,* p* = .002, η_p_^2^ = .21 (633 vs. 653 ms)	*F*(1, 39) = 11.69,* p* = .001, η_p_^2^ = .23 (634 vs. 657 ms)
				MT	*F*(1, 39) = 2.90, *p* = .097, η_p_^2^ = .07	*F* < 1
				%CC	*t*(39) = 2.78, *p* = .008, *d*_*z*_ = 0.44 (8.6% vs. 11.8%)	
Tonn et al. (2023)						
Exp. 1	Can REC effects be observed for actions that prevent (instead of produce) sensory effects? Typical actions that produce sensory effects serve as baseline condition	REC × effect-preventing vs. effect-producing actions	312	AUC	*F*(1, 42) = 13.98, *p* = .001, η_p_^2^ = .25 (3.5 × 10^3^ vs. 6.2 × 10^3^ px^2^)	*F* < 1
				MAD	*F*(1, 42) = 17.48,* p* < .001, η_p_^2^ = .29 (16.5 vs. 30.6 px)	*F* < 1
				IT	*F*(1, 42) = 34.62, *p* < .001, η_p_^2^ = .45 (632 vs. 673 ms)	*F*(1, 42) = 48.99, *p* < .001, η_p_^2^ = .54 (634 vs. 683 ms)
				MT	*F*(1, 42) = 47.93, *p* < .001, η_p_^2^ = .53 (506 vs. 562 ms)	*F*(1, 42) = 30.12, *p* < .001, η_p_^2^ = .42 (481 vs. 524 ms)
				%CC	*t*(42) = 5.01, *p* < .001, *d*_*z*_ = 0.76 (13.7% vs. 21.2%)	
Exp. S1	In contrast to Exp. 1, to-be-produced and to-be-prevented effects were no longer associated with monetary gains/losses	REC × effect-preventing vs. effect-producing actions	312	AUC	*F*(1, 40) = 14.91,* p* < .001, η_p_^2^ = .27 (3.7 × 10^3^ vs. 6.5 × 10^3^ px^2^)	*F* < 1
				MAD	*F*(1, 40) = 17.08,* p* < .001, η_p_^2^ = .30 (16.9 vs. 30.9 px)	*F* < 1
				IT	*F*(1, 40) = 40.36, *p* < .001, η_p_^2^ = .50 (588 vs. 635 ms)	*F*(1, 40) = 52.36, *p* < .001, η_p_^2^ = .57 (591 vs. 646 ms)
				MT	*F*(1, 40) = 31.24, *p* < .001, η_p_^2^ = .44 (423 vs. 461 ms)	*F*(1, 40) = 9.30, *p* = .004, η_p_^2^ = .19 (401 vs. 422 ms)
				%CC	*t*(40) = 6.14, *p* < .001, *d*_*z*_ = 0.96 (12.7% vs. 21.9%)	
Exp. S2	In contrast to Exp. 2, unsuccessful effect-preventing actions were no longer associated with unpleasant auditive effects	REC × effect-preventing vs. effect-producing actions	312	AUC	*F*(1, 40) = 28.88,* p* < .001, η_p_^2^ = .42 (3.6 × 10^3^ vs. 7.1 × 10^3^ px^2^)	*F* < 1
				MAD	*F*(1, 40) = 30.86,* p* < .001, η_p_^2^ = .44 (18.0 vs. 35.4 px)	*F* < 1
				IT	*F*(1, 40) = 96.42, *p* < .001, η_p_^2^ = .71 (538 vs. 579 ms)	*F*(1, 40) = 72.79, *p* < .001, η_p_^2^ = .65 (541 vs. 589 ms)
				MT	*F*(1, 40) = 85.02, *p* < .001, η_p_^2^ = .68 (449 vs. 491 ms)	*F*(1, 40) = 28.89, *p* < .001, η_p_^2^ = .42 (423 vs. 448 ms)
				%CC	*t*(40) = 8.55, *p* < .001, *d*_*z*_ = 1.34 (15.9% vs. 25.3%)	
Exp. S3	Effect-producing actions only	REC	156	AUC	*t*(45) = 3.84, *p* < .001, *d*_*z*_ = 0.57 (6.1 × 10^3^ vs. 9.1 × 10^3^ px^2^)	*t*(45) = 1.44, *p* = .156, *d*_*z*_ = 0.21
				MAD	*t*(45) = 4.12, *p* < .001, *d*_*z*_ = 0.61 (26.8 vs. 43.0 px)	*t*(45) = 1.62, *p* = .111, *d*_*z*_ = 0.24
				IT	*t*(45) = 5.56, *p* < .001, *d*_*z*_ = 0.82 (573 vs. 609 ms)	*t*(45) = 5.23, *p* < .001, *d*_*z*_ = 0.77 (580 vs. 621 ms)
				MT	*t*(45) = 7.06, *p* < .001, *d*_*z*_ = 1.04 (506 vs. 561 ms)	*t*(45) = 4.16, *p* < .001, *d*_*z*_ = 0.61 (475 vs. 515 ms)
				%CC	*t*(45) = 5.88, *p* < .001, *d*_*z*_ = 0.87 (17.3% vs. 25.4%)	

*DV *dependent variable; *REC* response effect compatibility; *IT *initiation time, *MT *movement time; *AUC *area under the curve; *MAD *maximum absolute distance; *%CC *percentage of trials that were classified by the cutoff criterion and excluded as multistep movements. For significant differences, descriptive values for the compatible (first descriptive value) and incompatible (second descriptive value) condition are provided

^a^Number of trials denotes the total number of trials for each participant, prior to any exclusions. In all experiments, participants worked through an equal amount of compatible and incompatible trials

^b^For brevity, only the effect of compatibility is reported, and influences of all other experimental factors are omitted. Thus, in multifactorial designs, *F*-statistics for the main effect of compatibility are reported, whereas in unifactorial designs, *t*-statistics are reported

^c^ Sensory compatibility denotes whether the location of an effects corresponds to the movement direction (i.e., “typical” spatial dimensional overlap as in the other experiments). Affective compatibility denotes whether a positive or negative event must be approached by the movement

^d^We omitted the factor “REC in the previous trial”’ and treated the data like a unifactorial design

Using all available data, a compatibility effect was observed for all four measures in Experiment 2 of Pfister et al. (2014). Compatible actions had smaller AUCs (-0.1 × 10^3^ vs. 2.7 × 10^3^ px^2^), *t*(19) = 3.59, *p* = .002, *d*_*z*_ = 0.80, smaller MADs (0.8 vs. 15.6 px), *t*(19) = 3.94, *p* = .001, *d*_*z*_ = 0.88, shorter ITs (639 vs. 693 ms), *t*(19) = 5.81, *p* < .001, *d*_*z*_ = 1.30, and shorter MTs (421 vs. 457 ms), *t*(19) = 3.12, *p* = .006, *d*_*z*_ = 0.70, than incompatible actions.

As expected, our cutoff criterion excluded fewer compatible than incompatible movements (9.8% vs. 17.5%), *t*(19) = 4.04, *p* = .001, *d*_*z*_ = 0.90. When applying this cutoff criterion, the compatibility effect vanished in the spatial measures of AUC, |*t*| < 1, and MAD, |*t*| < 1. In the timing measures, the test for the ITs remained significant, with compatible actions being initiated faster than incompatible actions (634 vs. 706 ms), *t*(19) = 6.31, *p* < .001, *d*_*z*_ = 1.41, whereas the effect in MTs vanished, |*t*| < 1.

**Fig. 5:**
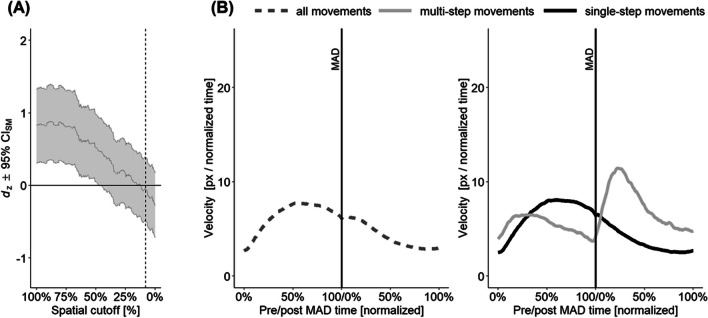
Results from Pfister et al. (2014), Experiment 2. (**A**) Compatibility effect in area under the curve (AUC) as a function of the used cutoff criterion. The x-axis indicates the allowed horizontal movement between the center of starting area and the center of the wrong target area, normalized to percentage. The y-axis indicates the standardized effect size. The dashed vertical line indicates the cutoff used in the text. (**B**) Velocity profiles for all movements (left), and movements classified with the cutoff criterion. The light-gray line depicts movements excluded from the analysis while the black line depicts movements remaining in the analysis. The solid vertical lines mark the point of maximal deviation from an ideal trajectory, with times on the x-axis normalized to percentage from start up this point to as well as from this point to reaching the target

Panel A of Fig. 5 shows that the compatibility effect remains non-significant even when our criterion is relaxed to permit movements to travel up to 40% of the horizontal distance towards the incorrect target area. Panel B of Fig. 5 illustrates that this simple cutoff criterion successfully classifies the movements into two distinct subgroups with markedly different velocity profiles: Single-step movements display a smooth velocity around the time of reaching MAD, whereas multi-step movements display a deceleration that is followed by a substantial acceleration around the time of reaching MAD.

## Discussion

This paper had two different aims. The first aim was to suggest an easy-to-implement method that separates two groups of mouse trajectories with different underlying processes. The second aim was to apply this method to published experiments within one exemplary field and to examine how the interpretation of previous data is affected by these different groups of trials.

To distinguish between smooth single-step movements and multi-step movements in which an initial, incomplete decision is revised along the movement trajectory, a vertical cutoff criterion was implemented. This spatial criterion is straightforward to implement and effectively separates both movement types, as evidenced by the velocity profile plots: While single-step movements exhibited rather smooth velocity profiles with high speeds at the point of maximum deviation, multi-step movements exhibited a pronounced deceleration before and rapid acceleration after the point of maximum deviation (Vogel et al., 2018). Thus, the binary classification (multistep: yes or no?) provided by our simple criterion suffices to illustrate the presence of (at least) two markedly different trajectory types that are mixed up when relying solely on average statistics.

In fact, the re-analyses of previous experiments revealed that a major proportion of the systematic variance in spatial measures resulted from multi-step movements, which constituted only a minor portion of the trials. In 10 out of 12 experiments, the compatibility effect in AUCs and MADs completely vanished after excluding movements starting in the wrong direction. This supports a recent speculation suggesting that these initial movements towards the wrong location may be the driving factor for influences on spatial trajectory markers in response-effect compatibility setups (Tonn et al., 2023), and therefore speaks against previous interpretations which ascribed these influences to a continuous activation of the anticipated perceptual effects (Hommel et al., 2017; Pfister et al., 2014). It is important to note that across all experiments, the response-effect compatibility effect for initiation times remained significant (and sometimes even increased in magnitude) after excluding trials with initial decision errors. Thus, while our analyses yielded no evidence for an influence on motor execution, temporal measures indicated a strong influence of response-effect compatibility on decision making.

But why differentiate between different types of movements and investigate where the compatibility effect originates from? The question of *whether* an anticipated action effect is represented is indeed not affected by this distinction. However, in the ideomotor framework, mouse-tracking was specifically employed to make precise inferences on *when* and *how* the anticipated action effect influences movements (Hommel et al., 2017; Pfister et al., 2014), that is, which action stages are affected by effect anticipations. In other words, observed influences were ascribed to a specific phase within a movement, to action execution, which in this context referred to the efferent activity that follows a completed decision. Thus, identifying the impact of movements initially starting into the wrong direction is critical because, in these trials, the decision phase was not completed before movement start. From our analyses we can conclude that the effects in the average trajectories do not primarily originate from movements starting with a completed decision towards the correct location. Therefore, the observed deviations in incompatible trajectories do not yield evidence for a continuous activation of the anticipated perceptual effects of the movement during its execution: This pattern mainly reflects decision errors during action selection instead.

Consequently, we suggest that researchers implement design features that hamper decision changes within the mouse-tracking paradigm or, alternatively, cross-validate results through other approaches when aiming to make inferences on movement execution rather than action selection. However, is it even possible to unequivocally separate action execution from decision making? At a broad level, one might argue that movements without changes-of-mind represent “pure” motor execution. On a more nuanced level, however, movements inherently involve a decision-making and planning component, as the system continuously “decides” whether (and how) to adjust the execution of an action. Consequently, a movement may contain decisional influences even when excluding all initial decision errors. Conversely, not all discontinuities (e.g., rapid speed or direction changes) necessarily indicate an erroneous initial decision but could likewise result from a correct decision where the execution failed at any point (e.g., due to muscle twitches). Nevertheless, in either scenario it can be argued that discontinuities in the movements reflect decision making while acting (Netick & Klapp, 1994; Vogel et al., 2024), irrespective of whether an erroneous decision or an erroneous execution is corrected.

Of course, mouse-tracking is not always utilized to make inferences on “pure” motor execution without leakage of decisional processes. Rather, various fields of research employ methods that aim at increasing (instead of decreasing) the temporal overlap of action selection and action execution to tightly couple cognitive and motor processes. Such approaches may include instructing participants to start moving swiftly (Freeman & Ambady, 2011; Hehman et al., 2015; Stolier & Freeman, 2016) or displaying imperative stimuli only after movement onset (e.g., Kieslich et al., 2020; Scherbaum & Kieslich, 2018; Schoemann et al., 2019). Thus, movements with incomplete or erroneous decisions are the primary research target there and should not be excluded from the analyses. However, as many studies are based on the assumption that cognitive processes manifest in the movement at the point in time where they occur (Dshemuchadse et al., 2013; Stillman et al., 2018), analyzing velocity profiles, as demonstrated here, provides vital new insights: It has recently been stated that interruptions in the form of pauses in the movement decouple the cognition-movement connection (Schoemann et al., 2021). Consequently, there are efforts to exclude such trials by eliminating the respective data points (e.g., Schonard et al., 2021) or by introducing design features that make pauses less likely, for example, by reducing the overall time limit (e.g., Boschet et al., 2022; Garcia-Guerrero et al., 2022). While this is a promising starting point towards more straightforward interpretations, it might only exclude a fraction of the movements where the cognition-movement connection is decoupled: Error research suggests that neural correlates of error processing can start even before the erroneous response is initiated (Yeung et al., 2004). Consequently, errors can be canceled extremely quickly or are even corrected on the fly (Foerster et al., 2022a, b). As error correction times are very short (e.g., Cooke & Diggles, 1984), the time until the corrective movement is initiated might be shorter than the time required to overcome the mass-inertia of the hand executing the erroneous movement. Therefore, the phases of decelerating the movement in the direction of the wrong response and accelerating the movement in the direction of the correct response might overlap, raising the question whether the two components are indeed always separated by a complete stop. Consequently, researchers are well advised to explicitly check not only for pauses, but also for dips in the velocity profiles because any kind of velocity change (e.g., a notable deceleration of the movement) might indicate a decoupling of the cognition-movement connection and thereby hide processes taking place. A more detailed look into the origin of observed effects regarding velocities within a movement, trajectories on a by-participant and by-trial level, and a specification of whether these effects depict errors or a deliberate strategy to postpone a decision (Wong & Haith, 2017), can advance the interpretations drawn from mouse-tracking experiments in various fields.

How do the current methodological considerations inform ideomotor theorizing? Influences of effect anticipations on action execution have not only been investigated by evidently metric movement trajectories, but also by metric aspects of seemingly discrete keypress movements such as duration or force of the executed keypress. This raises the question of whether the current results also have implications for the interpretation of these experiments. In other words, do experimental setups in which anticipated effects manifest in the parameters of executed actions generally reflect only decisional influences? We believe that this is not the case. In mouse-tracking setups, participants have ample time to correct an incorrect response during its execution. Most keypress experiments, however, do not provide this opportunity (notable exceptions are mainly found in literature on error processing; e.g., Crump & Logan, 2013; Rabbitt, 1966). Instead, participants usually know that the onset of a keypress immediately categorizes their movement as either correct or wrong. If an initially incorrect response exceeds the key’s activation threshold, the trial is directly classified as error, regardless of whether the correct key is pressed afterwards. In these designs, the onset of a keypress serves as a natural barrier beyond which a change of mind can no longer be implemented. Therefore, if anticipated effects still influence action execution after the onset of the keypress, this suggests that the observed influences go beyond a decisional component.

We recently investigated keypress durations (i.e., the time between pressing and releasing a key; Pfister et al., 2023; Shin et al., 2023) in such a design and indeed found the duration of keypresses to be biased towards the duration of irrelevant auditory effects (Tonn et al., 2023). Similarly, in a study investigating motor sequences, execution times (i.e., the time between the onsets of the first and the last keypress) showed assimilative influences of temporal effect anticipations (Brown et al., 2022). Together, these results suggest that response-effect compatibility effects in action execution are not generally driven by categorical decision making. Interestingly, actions have not only been reported to align with features of the ensuing effects, but also to diverge from them. For instance, contrast effects were observed when participants were specifically instructed to press a key for either a short or a long duration (Kunde, 2003), or with low or high force (Kunde et al., 2004; Thébault et al., 2020), resulting in tones of varying lengths or intensities. Unlike experiments that found assimilation effects, these experiments involved task-relevant action features. Thus, it is conceivable that for task-relevant features, participants intuitively counteracted the natural tendency to align their actions with the anticipated effect to prevent errors.

## Conclusion

Decision errors are pervasive in mouse-tracking studies. Understood in the context of a single response, such decision errors relate to a response that is initiated in a wrong direction but might be corrected later during the movement. If an experiment seeks to measure any influence of an experimental condition, such decision errors do not pose a concern. They are of substantial concern, however, if researchers intend to specifically investigate how completed decisions are put into motion: Not accounting for changes of mind during the execution of an action leads to erroneous interpretation of aggregate statistics. Thus, when investigating what movement kinematics reveal about human cognition, researchers are well advised to take full advantage of the rich information inherent in every single trajectory instead of interpreting the shape of average trajectories.

## Figures and Tables

**Fig. 6:**
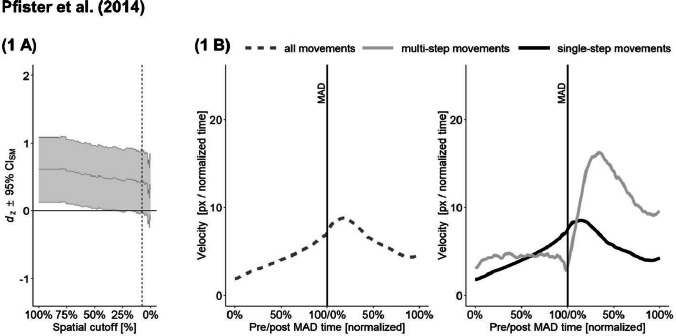
Results from Pfister et al. (2014), Experiment 1.** (A)** Compatibility effect in AUC as a function of the used cutoff criterion. The x-axis indicates the allowed horizontal movement between the center of starting area and the center of the wrong target area, normalized to percentage. The y-axis indicates the standardized effect size. The dashed vertical line indicates the cutoff used in the text. **(B)** Velocity profiles for all movements (left), and movements classified with the cutoff criterion. The light-grey line depicts movements excluded from the analysis while the black line depicts movements remaining in the analysis. The solid vertical lines mark the point of maximal deviation from an ideal trajectory, with times on the x-axis normalized to percentage from start up this point to as well as from this point to reaching the target

**Fig. 7:**
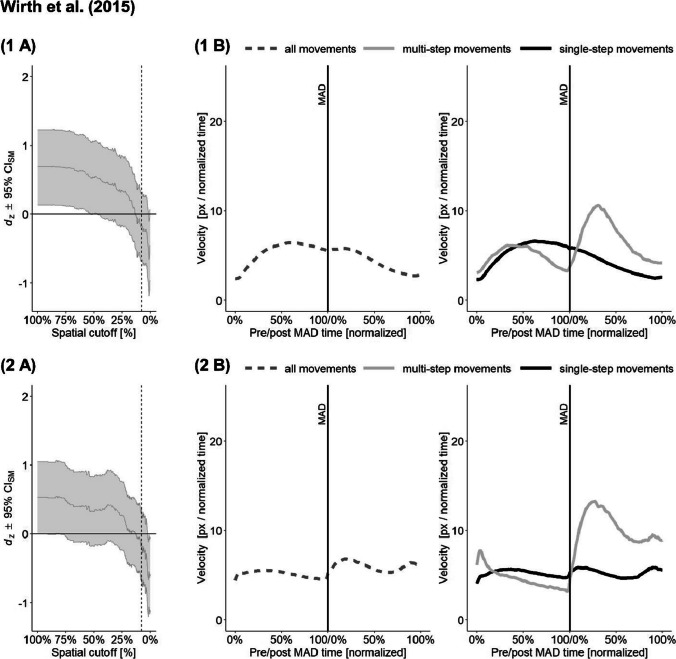
Results from Wirth et al. (2015), Experiment 1 and 2.** (A)** Compatibility effect in AUC as a function of the used cutoff criterion. The x-axis indicates the allowed horizontal movement between the center of starting area and the center of the wrong target area, normalized to percentage. The y-axis indicates the standardized effect size. The dashed vertical line indicates the cutoff used in the text. **(B)** Velocity profiles for all movements (left), and movements classified with the cutoff criterion. The light-grey line depicts movements excluded from the analysis while the black line depicts movements remaining in the analysis. The solid vertical lines mark the point of maximal deviation from an ideal trajectory, with times on the x-axis normalized to percentage from start up this point to as well as from this point to reaching the target

**Fig. 8:**
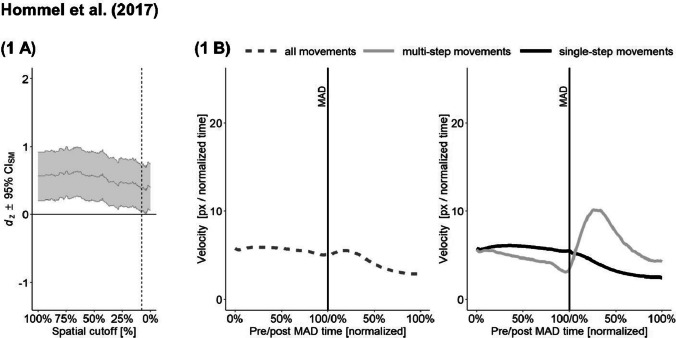
Results from Hommel et al. (2017).** (A)** Compatibility effect in AUC as a function of the used cutoff criterion. The x-axis indicates the allowed horizontal movement between the center of starting area and the center of the wrong target area, normalized to percentage. The y-axis indicates the standardized effect size. The dashed vertical line indicates the cutoff used in the text. **(B)** Velocity profiles for all movements (left), and movements classified with the cutoff criterion. The light-grey line depicts movements excluded from the analysis while the black line depicts movements remaining in the analysis. The solid vertical lines mark the point of maximal deviation from an ideal trajectory, with times on the x-axis normalized to percentage from start up this point to as well as from this point to reaching the target. Note that in this experiment, the data logging rate was substantially higher than the polling rate of the mouse. To prevent edge artifacts, data without updated position information had to be excluded via a custom script prior to time-normalization (for details see https://osf.io/hrpk6)

**Fig. 9:**
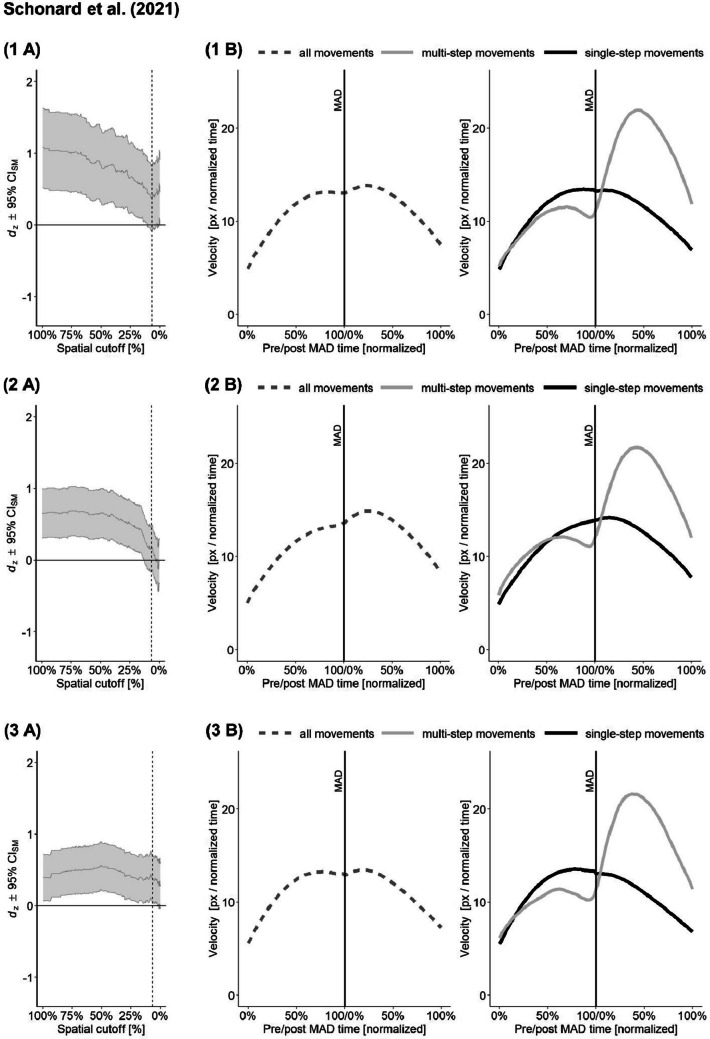
Results from Schonard et al. (2021), Experiment 1, 2, and 3.** (A)** Compatibility effect in AUC as a function of the used cutoff criterion. The x-axis indicates the allowed horizontal movement between the center of starting area and the center of the wrong target area, normalized to percentage. The y-axis indicates the standardized effect size. The dashed vertical line indicates the cutoff used in the text. **(B)** Velocity profiles for all movements (left), and movements classified with the cutoff criterion. The light-grey line depicts movements excluded from the analysis while the black line depicts movements remaining in the analysis. The solid vertical lines mark the point of maximal deviation from an ideal trajectory, with times on the x-axis normalized to percentage from start up this point to as well as from this point to reaching the target

**Fig 10:**
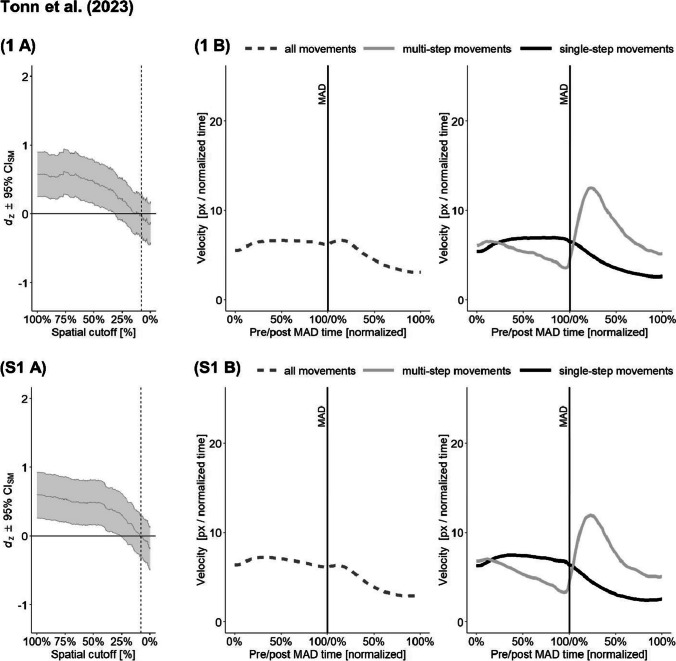
Results from Tonn et al. (2023), Experiment 1, S1. **(A)** Compatibility effect in AUC as a function of the used cutoff criterion. The x-axis indicates the allowed horizontal movement between the center of starting area and the center of the wrong target area, normalized to percentage. The y-axis indicates the standardized effect size. The dashed vertical line indicates the cutoff used in the text. **(B)** Velocity profiles for all movements (left), and movements classified with the cutoff criterion. The light-grey line depicts movements excluded from the analysis while the black line depicts movements remaining in the analysis. The solid vertical lines mark the point of maximal deviation from an ideal trajectory, with times on the x-axis normalized to percentage from start up this point to as well as from this point to reaching the target

**Fig. 11:**
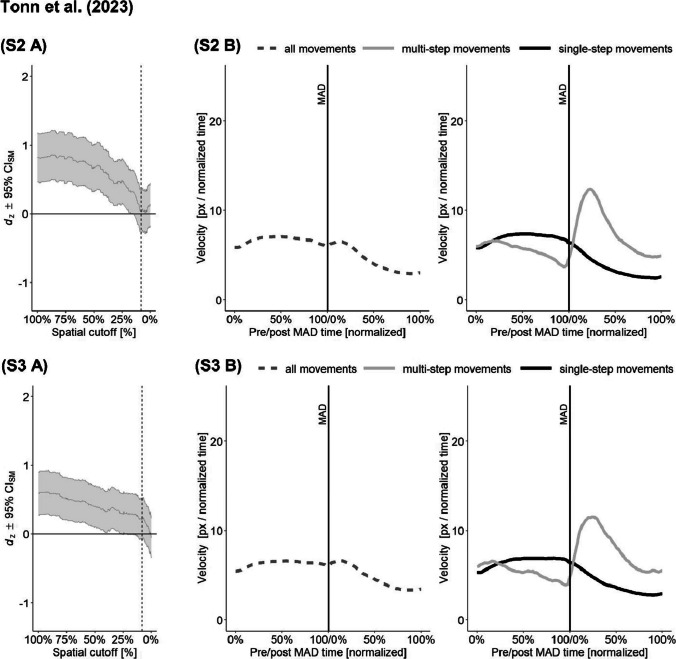
Results from Tonn et al. (2023), Experiment S2, S3.** (A)** Compatibility effect in AUC as a function of the used cutoff criterion. The x-axis indicates the allowed horizontal movement between the center of starting area and the center of the wrong target area, normalized to percentage. The y-axis indicates the standardized effect size. The dashed vertical line indicates the cutoff used in the text. **(B)** Velocity profiles for all movements (left), and movements classified with the cutoff criterion. The light-grey line depicts movements excluded from the analysis while the black line depicts movements remaining in the analysis. The solid vertical lines mark the point of maximal deviation from an ideal trajectory, with times on the x-axis normalized to percentage from start up this point to as well as from this point to reaching the target

## Data Availability

All data, analyses, and figure scrips are publicly available via the Open Science Framework (https://osf.io/hrpk6). Preregistrations are based on the original authors’ publications. Three experiments in the publication of Tonn et al. (2023) were preregistered.

## Appendix

This appendix discusses the usage of an additional velocity criterion and provides the complete results and visualizations for all experiments which were not described in detail in the main body.

### Velocities as additional criterion

Although spatial information is an important characteristic of the multi-step movements we aim to exclude (i.e., trials that initially start into the wrong direction), a spatial cutoff-criterion might fail to identify some multi-step movements and conversely, might classify some smooth movements as multi-step. To address this, we supplemented our analyses with velocity profile plots and visualizations of the empirical effect as function of different cutoff values. The velocity profile plots demonstrate that our criterion can partition the movements into two disjunct sets, each displaying the acceleration characteristics expected of either multi-step or single-step movements. Additionally, the visualizations of the adjusted cutoff-values show that the pattern of results remains stable regardless of whether the cutoff is positioned exactly at the border of the starting area. Although such adjustments do not enhance the inherent accuracy of the classifier itself, making the cutoff more lenient or more stringent can alter the balance between false-positives and false-negatives. Improving overall accuracy could be possible by, for example, using the velocity around the point of reaching MAD as an additional criterion.

In the current re-analyses, however, this approach comes with a substantial drawback: Because the velocities around the point of reaching the MAD were used as means to validate the spatial criterion, using them as additional quantitative criterion would undermine the validation process. Yet, if another validation method is available – such as manual labeling of movements – or if validation is not a major concern, then velocity profiles could indeed serve as supplementary quantitative criterion to enhance classification accuracy. Specifically, we propose using the velocity at the point of reaching MAD, relative to some characteristic of the trial’s velocity distribution, such as maximum or median speed. Similarly, exploring other movement characteristics around the point of reaching MAD, for example the rate of change in movement direction (angular velocity), could also be beneficial. Incorporating multiple criteria into an ensemble classifier might improve overall categorization performance, potentially offering a more accurate approach for distinguishing between multi-step and single-step movements. For researchers particularly interested in the methodology of mouse tracking per se, velocity-based classifications could be of substantial benefit. We added an exemplary script to the OSF repository that, for the main experiment of this article, compares our spatial criterion with a criterion taking velocity around MAD into account.

Nonetheless, we believe that the main strength and unique advantage of our current criterion lies in its simplicity, especially when compared to other methods that are more advanced and thus more difficult to implement. In our view, its performance is quite satisfying, and the additional effort required for more complex methods may pose a significant barrier for researchers that consider mouse tracking to be just one part of a broader toolkit for investigating other research areas. For such researchers, our criterion likely provides a good balance of efficiency and effectiveness.

### Pfister et al. (2014)

Using all available data, a compatibility effect was observed for all four measures. Compatible actions had smaller AUCs (15.8 × 10^3^ vs. 16.9 × 10^3^ px^2^), *t*(19) = 2.75, *p* = .013, *d*_*z*_ = 0.61, smaller MADs (64.1 vs. 68.1 px), *t*(19) = 2.58, *p* = .018, *d*_*z*_ = 0.58, shorter ITs (652 vs. 672 ms), *t*(19) = 2.33, *p* = .031, *d*_*z*_ = 0.52, and shorter MTs (590 vs. 628 ms), *t*(19) = 3.17, *p* = .005, *d*_*z*_ = 0.71, than incompatible actions.

As expected, our cutoff criterion excluded fewer compatible than incompatible movements (5.9% vs. 10.3%), *t*(19) = 3.19, *p* = .005, *d*_*z*_ = 0.71. When applying our cutoff criterion, the compatibility effect vanished in the spatial measures of AUC, *t*(19) = 1.89, *p* = .073, *d*_*z*_ = 0.42, and MAD, *t*(19) = 1.17, *p* = .258, *d*_*z*_ = 0.26. In contrast, it remained present only in the timing measures: Compatible actions had shorter ITs (654 vs. 672 ms), *t*(19) = 2.17, *p* = .043, *d*_*z*_ = 0.49, and shorter MTs (578 vs. 598 ms), *t*(19) = 2.77, *p* = .012, *d*_*z*_ = 0.62, than incompatible actions.

Figure 6 shows the results for Experiment 1 in Pfister et al. (2014).

### Wirth et al. (2015)

Using all available data of Experiment 1, a compatibility effect was observed for all four measures. Compatible actions had smaller AUCs (0.7 × 10^3^ vs. 2.8 × 10^3^ px^2^), *F*(1, 15) = 7.63, *p* = .015, η_p_^2^ = .34, smaller MADs (4.2 vs. 15.0 px), *F*(1, 15) = 8.19, *p* = .012, η_p_^2^ = .35, shorter ITs (880 vs. 950 ms), *F*(1, 15) = 26.50, *p* < .001, η_p_^2^ = .64, and shorter MTs (375 vs. 399 ms), *F*(1, 15) = 13.58, *p* = .002, η_p_^2^ = .48, than incompatible actions. Note that the original paper did not analyze MAD.

As expected, our cutoff criterion excluded fewer compatible than incompatible movements (9.4% vs. 17.1%), *t*(15) = 4.30, *p* = .001, *d*_*z*_ = 1.08. When applying our cutoff criterion, the compatibility effect vanished in the spatial measures of AUC, *F* < 1, and MAD, *F* < 1. In the timing measures, the test for the ITs remained significant, with compatible actions initiated faster than incompatible actions (879 vs. 950 ms), *F*(1, 15) = 27.17, *p* < .001, η_p_^2^ = .64, whereas the effect in MTs vanished, *F*(1, 15) = 1.70, *p* = .212, η_p_^2^ = .10.

Using all available data of Experiment 2, no effect was observed for AUCs, *F*(1, 15) = 4.53, *p* = .050, η_p_^2^ = .23, but compatible actions had smaller MADs than incompatible actions (22.8 vs. 27.9 px), *F*(1, 15) = 6.40, *p* = .023, η_p_^2^ = .30. In the timing measures, ITs did not differ, *F*(1, 15) = 3.55, *p* = .079, η_p_^2^ = .19, but MTs were shorter with compatible than with incompatible actions (1015 vs. 1091 ms), *F*(1, 15) = 9.78, *p* = .007, η_p_^2^ = .39.

As expected, our cutoff criterion excluded fewer compatible than incompatible movements (13.0% vs. 16.6%), *t*(15) = 2.26, *p* = .039, *d*_*z*_ = 0.56. When applying our cutoff criterion, the compatibility effect vanished in the spatial measures of AUC, *F* < 1, and MAD, *F*(1, 15) = 1.00, *p* = .334, η_p_^2^ = .06. In the timing measures, ITs did not differ, *F*(1, 15) = 3.87, *p* = .068, η_p_^2^ = .21, but MTs were shorter with compatible than with incompatible actions (988 vs. 1053 ms), *F*(1, 15) = 7.66, *p* = .014, η_p_^2^ = .34.

Figure 7 shows the results for Experiment 1 and Experiment 2 in Wirth et al. (2015).

### Hommel et al. (2017)

Using all available data, a compatibility effect was observed for all four measures. Compatible actions had smaller AUCs (3.2 × 10^3^ vs. 5.3 × 10^3^ px^2^), *F*(1, 34) = 10.41, *p* = .003, η_p_^2^ = .23, smaller MADs (17.7 vs. 27.6 px), *F*(1, 34) = 9.77, *p* = .004, η_p_^2^ = .22, shorter ITs (496 vs. 507 ms), *F*(1, 34) = 7.19, *p* = .011, η_p_^2^ = .17, and shorter MTs (339 vs. 347 ms), *F*(1, 34) = 5.69, *p* = .023, η_p_^2^ = .14, than incompatible actions.

As expected, our cutoff criterion excluded fewer compatible than incompatible movements (16.5% vs. 22.3%), *t*(34) = 4.82, *p* < .001, *d*_*z*_ = 0.81. In this experiment, the pattern of significance remained the same after applying our cutoff criterion: Compatible actions still had smaller AUCs (-0.7 × 10^3^ vs. 0.3× 10^3^ px^2^), *F*(1, 34) = 5.45, *p* = .026, η_p_^2^ = .14, smaller MADs (-2.4 vs. 1.5 px), *F*(1, 34) = 4.76, *p* = .036*,* η_p_^2^ = .12, shorter ITs (497 vs. 507 ms), *F*(1, 34) = 5.38, *p* = .027, η_p_^2^ = .14, and shorter MTs (317 vs. 324 ms), *F*(1, 34) = 5.24, *p* = .028, η_p_^2^ = .13, than incompatible actions.

Figure 8 shows the results for Hommel et al. (2017).

### Schonard et al. (2021)

Using all available data of Experiment 1, a compatibility effect was found for spatial measures. Compatible actions had smaller AUCs (22.7 × 10^3^ vs. 33.7 × 10^3^ px^2^), *F*(1, 19) = 23.22, *p* < .001, η_p_^2^ = .55, and smaller MADs (32.4 vs. 51.7 px), *F*(1, 19) = 23.00, *p* < .001, η_p_^2^ = .55, than incompatible actions. In the timing measures, ITs did not differ, *F*(1, 19) = 3.77, *p* = .067, η_p_^2^ = .17, but MTs were shorter with compatible than with incompatible actions (544 vs. 572 ms), *F*(1, 19) = 30.65, *p* < .001, η_p_^2^ = .62.

As expected, our cutoff criterion excluded fewer compatible than incompatible movements (7.1% vs. 16.1%), *t*(19) = 5.52, *p* < .001, *d*_*z*_ = 1.23. When applying our cutoff criterion, the compatibility effect vanished for the spatial measures of AUC, *F*(1, 19) = 3.05, *p* = .097*,* η_p_^2^ = .14, and MAD, *F*(1, 19) = 3.02, *p* = .098*,* η_p_^2^ = .14. In the timing measures, ITs did not differ, *F*(1, 19) = 3.50, *p* = .077, η_p_^2^ = .16, but MTs were shorter with compatible than with incompatible actions (535 vs. 552 ms), *F*(1, 19) = 6.43, *p* = .020, η_p_^2^ = .25.

Using all available data of Experiment 2, a compatibility effect was found for all four measures. Compatible actions had smaller AUCs (63.2 × 10^3^ vs. 72.7 × 10^3^ px^2^), *t*(39) = 4.37, *p* < .001, *d*_*z*_ *=* 0.69, smaller MADs (92.5 vs. 108.1 px), *t*(39) = 4.22, *p* < .001, *d*_*z*_ = 0.67, shorter ITs (602 vs. 645 ms), *t*(39) = 5.15, *p* < .001, *d*_*z*_ = 0.81, and shorter MTs (633 vs. 661 ms), *t*(39) = 4.75, *p* < .001, *d*_*z*_ = 0.75, than incompatible movements.

As expected, our cutoff criterion excluded fewer compatible than incompatible movements (11.1% vs. 17.4%), *t*(39) = 5.11, *p* < .001, *d*_*z*_ = 0.81. When applying our cutoff criterion, the compatibility effect vanished in the spatial measures of AUC, *t*(39) = 1.02, *p* = .312, *d*_*z*_ = 0.16, and MAD, |*t*| < 1. In contrast, it remained present in the timing measures: Compatible actions had shorter ITs (604 vs. 658 ms), *t*(39) = 5.80, *p* < .001, *d*_*z*_ = 0.92, and shorter MTs (613 vs. 628 ms), *t*(39) = 3.14, *p* = .003, *d*_*z*_ = 0.50, than incompatible actions.

Using all available data of Experiment 3, a compatibility effect was found for the spatial measures. Compatible actions had smaller AUCs (13.8 × 10^3^ vs. 19.3 × 10^3^ px^2^), *F*(1, 39) = 8.89, *p =* .005, η_p_^2^ *= .*19, and smaller MADs (20.7 vs. 30.3 px), *F*(1, 39) = 8.63, *p* = .006, η_p_^2^ = .18, than incompatible actions. In the timing measures, ITs were shorter for compatible actions than for incompatible actions (633 vs. 653 ms), *F*(1, 39) = 10.66, *p* = .002, η_p_^2^ = .21, but MTs did not differ, *F*(1, 39) = 2.90, *p* = .097, η_p_^2^ = .07.

As expected, our cutoff criterion excluded fewer compatible than incompatible movements (8.6% vs. 11.8%), *t*(39) = 2.78, *p* = .008, *d*_*z*_ = 0.44. In this experiment, the pattern of significance remained the same after applying our cutoff criterion: Compatible actions still had smaller AUCs (7.2 × 10^3^ vs. 9.7 × 10^3^ px^2^), *F*(1, 39) = 5.42, *p =* .025, η_p_^2^ *= .*12, smaller MADs (9.5 vs. 13.0 px), *F*(1, 39) = 4.94, *p* = .032, η_p_^2^ = .11, and shorter ITs (634 vs. 657 ms), *F*(1, 39) = 11.69, *p* = .001, η_p_^2^ = .23, than incompatible actions, and MTs again did not differ, *F* < 1.

Figure 9 shows the results for Experiment 1, Experiment 2, and Experiment 3 in Schonard et al. (2021).

### Tonn et al. (2023)

Using all available data of Experiment 1, a compatibility effect was found for all four measures. Compatible actions had smaller AUCs (3.5 × 10^3^ vs. 6.2 × 10^3^ px^2^), *F*(1, 42) = 13.98, *p =* .001, η_p_^2^ *= .*25, smaller MADs (16.5 vs. 30.6 px), *F*(1, 42) = 17.48, *p* < .001, η_p_^2^ = .29, shorter ITs (632 vs. 673 ms), *F*(1, 42) = 34.62, *p* < .001, η_p_^2^= .45, and shorter MTs (506 vs. 562 ms), *F*(1, 42) = 47.93, *p* < .001, η_p_^2^ = .53, than incompatible actions.

As expected, our cutoff criterion excluded fewer compatible than incompatible movements (13.7% vs. 21.2%), *t*(42) = 5.01, *p* < .001, *d*_*z*_ = 0.76. When applying our cutoff criterion, the compatibility effect vanished in the spatial measures of AUCs, *F* < 1, and MAD, *F* < 1. In contrast, it remained present in the timing measures: Compatible actions had shorter ITs (634 vs. 683 ms), *F*(1, 42) = 48.99, *p* < .001, η_p_^2^ = .54, and shorter MTs (481 vs. 524 ms), *F*(1, 42) = 30.12, *p* < .001, η_p_^2^ = .42, than incompatible actions.

Using all available data of Experiment S1, a compatibility effect was found for all four measures. Compatible actions had smaller AUCs (3.7 × 10^3^ vs. 6.5 × 10^3^ px^2^), *F*(1, 40) = 14.91, *p* < .001, η_p_^2^ *=* .27, smaller MADs (16.9 vs. 30.9 px), *F*(1, 40) = 17.08, *p* < .001, η_p_^2^ = .30, shorter ITs (588 vs. 635 ms), *F*(1, 40) = 40.36, *p* < .001, η_p_^2^ = .50, and shorter MTs (423 vs. 461 ms), *F*(1, 40) = 31.24, *p* < .001, η_p_^2^ = .44, than incompatible actions.

As expected, our cutoff criterion excluded fewer compatible than incompatible movements (12.7% vs. 21.9%), *t*(40) = 6.14, *p* < .001, *d*_*z*_ = 0.96. When applying our cutoff criterion, the compatibility effect vanished in the spatial measures of AUC, *F* < 1, and MAD, *F* < 1. In contrast, it remained present in the timing measures: Compatible actions had shorter ITs (591 vs. 646 ms), *F*(1, 40) = 52.36, *p* < .001, η_p_^2^ = .57, and shorter MTs (401 vs. 422 ms), *F*(1, 40) = 9.30, *p* = .004, η_p_^2^ = .19, than incompatible actions.

Using all available data of Experiment 2, a compatibility effect was found for all four measures. Compatible actions had smaller AUCs (3.6 × 10^3^ vs. 7.1 × 10^3^ px^2^), *F*(1, 40) = 28.88, *p* < .001, η_p_^2^ *=* .42, smaller MADs (18.0 vs. 35.4 px), *F*(1, 40) = 30.86, *p* < .001, η_p_^2^ = .44, shorter ITs (538 vs. 579 ms), *F*(1, 40) = 96.42, *p* < .001, η_p_^2^ = .71, and shorter MTs (449 vs. 491 ms), *F*(1, 40) = 85.02, *p* < .001, η_p_^2^ = .68, than incompatible trials.

As expected, our cutoff criterion excluded fewer compatible than incompatible movements (15.9% vs. 25.3%), *t*(40) = 8.55, *p* < .001, *d*_*z*_ = 1.34. When applying our cutoff criterion, the compatibility effect vanished in the spatial measures of AUC, *F* < 1, and MAD, *F* < 1. In contrast, it remained present in the timing measures: Compatible actions had shorter ITs (541 vs. 589 ms), *F*(1, 40) = 72.79, *p* < .001, η_p_^2^ = .65, and shorter MTs (423 vs. 448 ms), *F*(1, 40) = 28.89, *p* < .001, η_p_^2^ = .42, than incompatible actions.

Using all available data of Experiment S3, a compatibility effect was found for all four measures. Compatible actions had smaller AUCs (6.1 × 10^3^ vs. 9.1 × 10^3^ px^2^), *t*(45) = 3.84, *p* < .001, *d*_*z*_ = 0.57, smaller MADs (26.8 vs. 43.0 px), *t*(45) = 4.12, *p* < .001, *d*_*z*_ = 0.61, shorter ITs (573 vs. 609 ms), *t*(45) = 5.56, *p* < .001, *d*_*z*_ = 0.82, and shorter MTs (506 vs. 561 ms), *t*(45) = 7.06, *p* < .001, *d*_*z*_ = 1.04, than incompatible movements.

As expected, our cutoff criterion excluded fewer compatible than incompatible movements (17.3% vs. 25.4%), *t*(45) = 5.88, *p* < .001, *d*_*z*_ = 0.87. When applying our cutoff criterion, the compatibility effect vanished in the spatial measures of AUC, *t*(45) = 1.44, *p* = .156, *d*_*z*_ = 0.21, and MAD, *t*(45) = 1.62, *p* = .111, *d*_*z*_ = 0.24. In contrast, it remained present in the timing measures: Compatible actions had shorter ITs (580 vs. 621 ms), *t*(45) = 5.23, *p* < .001, *d*_*z*_ = 0.77, and shorter MTs (475 vs. 515 ms), *t*(45) = 4.16, *p* < .001, *d*_*z*_ = 0.61, than incompatible actions.

Figure 10 shows the results for Experiment 1 and Experiment S1 in Tonn et al. (2023).

Figure 11 shows the results for Experiment S2 and Experiment S3 in Tonn et al. (2023).

## Footnotes

1 Another simple spatial cutoff is related to the measure of x-flips, i.e., the number of directional changes along the x-axis (Dale & Duran, 2011; Duran et al., 2010). Movements with many x-flips are commonly multi-step movements.
